# Supplementary material for: Transcriptome Analysis Reveals the AhR, Smad2/3, and HIF-1α Pathways as the Mechanism of Ochratoxin A Toxicity in Kidney Cells
Source: Toxins (Basel). 2021 Mar 6;13(3):190. doi: 10.3390/toxins13030190 (PMC7999264; doi:10.3390/toxins13030190)
Supplement: Supplementary file 1 [file toxins-13-00190-s001.pdf]

Supplementary Materials: Transcriptome Analysis Reveals the AhR, Smad2/3, and HIF-1α Pathways as the Mechanism of Ochratoxin A Toxicity in Kidney Cells

Min Cheol Pyo, In-Geol Choi and Kwang-Won Lee

Table S1. Human qPCR primer sequences used in the experiments.

Origin	Marker	Sequence	
Human	AhR	Sense	5′-TGG ACA AGG AAT TGA AGA AGC-3′
		Antisense	5′-AAA GGA GAG TTT TCT GGA GGA A-3′
	Smad2	Sense	5′-ATG TCG TCC ATC TTG CCA TTC-3′
		Antisense	5′-AAC CGT CCT GTT TTC TTT AGC TT-3′
	Smad3	Sense	5′-ATC TAC TGC CGC CTG TGG-3′
		Antisense	5′-TCT CTG GTA GTG GTA GGG ATT C-3′
	HIF-1α	Sense	5′-AGC GAC AGA TAA CAC GTT AGG GCT-3′
		Antisense	5′-AGC CTC ACC AAA CAG AGC AGG AAA-3′
	E-cadherin	Sense	5′-ATT TTT CCC TCG ACA CCC GAT-3′
		Antisense	5′-TCC CAG GCG TAG ACC AAG A-3′
	Fibronectin	Sense	5′-CTG GCC GAA AAT ACA TTG TAA A-3′
		Antisense	5′-CCA CAG TCG GGT CAG GAG-3′
	Bax	Sense	5′-TCA GGA TGC GTC CAC CAA GAA G-3′
		Antisense	5′-TGT GTC CAC GGC GGC AAT CAT C-3′
	Bcl-2	Sense	5′-ATG TGT GTG GAG AGC GTC AA-3′
		Antisense	5′-CAG GAG AAA TCA AAC AGA GGC-3′
	Caspase 3	Sense	5′-GGA AGC GAA TCA ATG GAC TCT GG-3′
		Antisense	5′-GCA TCG ACA TCT GTA CCA GAC C-3′
	Caspase 9	Sense	5′-GTT TGA GGA CCT TCG ACC AGC T-3′
		Antisense	5′-CAA CGT ACC AGG AGC CAC TCT T-3′
	KIM-1	Sense	5′-GAA GTG GCT ACT GGT TCA TGG-3′
		Antisense	5′-ACG ACT GTT CGA ACG AGC AC-3′
	β-actin	Sense	5′-AGC GAG CAT CCC CCA AAG TT-3′
		Antisense	5′-GGG CAC GAA GGC TCA TCA TT-3′
